# Spatial immune profiling of glioblastoma identifies an inflammatory, perivascular phenotype associated with longer survival

**DOI:** 10.1007/s00401-023-02617-6

**Published:** 2023-08-13

**Authors:** Hans-Georg Wirsching, Jörg Felsberg, Michael Prummer, Vlad Moisoiu, Roxanne Lourman, Caroline Hertler, Michelle Antonios, Patrick J. Cimino, Patrick Roth, Thierry Gorlia, Robert M. Prins, Timothy Cloughesy, Patrick Y. Wen, Eric C. Holland, Guido Reifenberger, Michael Weller

**Affiliations:** 1 Department of Neurology, University Hospital and University of Zurich, Frauenklinikstrasse 26, CH-8091 Zurich, Switzerland; 2 Institute of Neuropathology, Medical Faculty, Heinrich Heine University and University Hospital Düsseldorf, Düsseldorf, Germany; 3 NEXUS Personalized Health Technologies and Swiss Institute of Bioinformatics, ETH Zurich, Zurich, Switzerland; 4 Department of Radiation Oncology, Competence Center for Palliative Care, University Hospital and University of Zurich, Zurich, Switzerland; 5 Surgical Neurology Branch, National Institute of Neurological Disorders and Stroke, National Institutes of Health, Bethesda, MD USA; 6 European Organization for Research and Treatment of Cancer, Brussels, Belgium; 7 Department of Neurosurgery, University of California Los Angeles (UCLA), Los Angeles, CA USA; 8 Center for Neuro-Oncology, Dana-Farber Cancer Institute and Harvard Medical School, Boston, MA USA; 9 Human Biology Division, Fred Hutchinson Cancer Center, Seattle, WA USA; 10 Partner Site Essen/Düsseldorf, German Cancer Consortium (DKTK), Düsseldorf, Germany

The historic term “glioblastoma multiforme” reflects the heterogeneity of histopathologic compartments in malignant gliomas [1]. Prominent and disease-defining histopathologic features of glioblastoma, the most common type of glioma, include microvascular proliferation and necrosis, the latter often with perinecrotic palisading of tumor cells [3]. These features are thought to result from a vicious cycle between dysfunctional, clotting blood vessels and perinecrotic areas where hypoxia and low pH drive an aberrant pro-angiogenic response, resulting in more dysfunctional blood vessels [6]. The interconversion between both compartments results in spatially resolved niches, where metabolically driven myeloid cell and tumor cell subpopulations interact [5]. Here, we have analyzed spatial immune profiles from clinically and molecularly well-annotated glioblastoma patient samples to explore clinical implications of spatial immune phenotyping, including the abundance of immunotherapy targets, and associations of spatial immune profiles with outcome. Patients and methods are detailed in supplementary Note S1.

First, we analyzed 360 anatomically defined glioblastoma regions of interest by spatial immune profiling utilizing a panel of 28 DNA bar-coded antibodies to quantify immune cell types and immunotherapy targets in perivascular, perinecrotic and cell-dense tumor regions (Fig. 1a, Table S1). Regions of interest were defined in 30 tissue sections from 20 patients diagnosed with glioblastoma according to the 2021 World Health Organization classification of central nervous system tumors [3], including paired samples from untreated primary tumors at diagnosis and recurrent tumors after standard chemoradiotherapy with temozolomide (paired sample cohort, PSC) of 10 patients from the central nervous systems tumor tissue bank Dusseldorf, and 10 samples from untreated primary tumors of patients included in the EORTC 1419 ETERNITY study, who survived for more than 5 years after diagnosis (long-term survivors, LTS, Figure S1, Table S2, [2]).

**Fig. 1:**
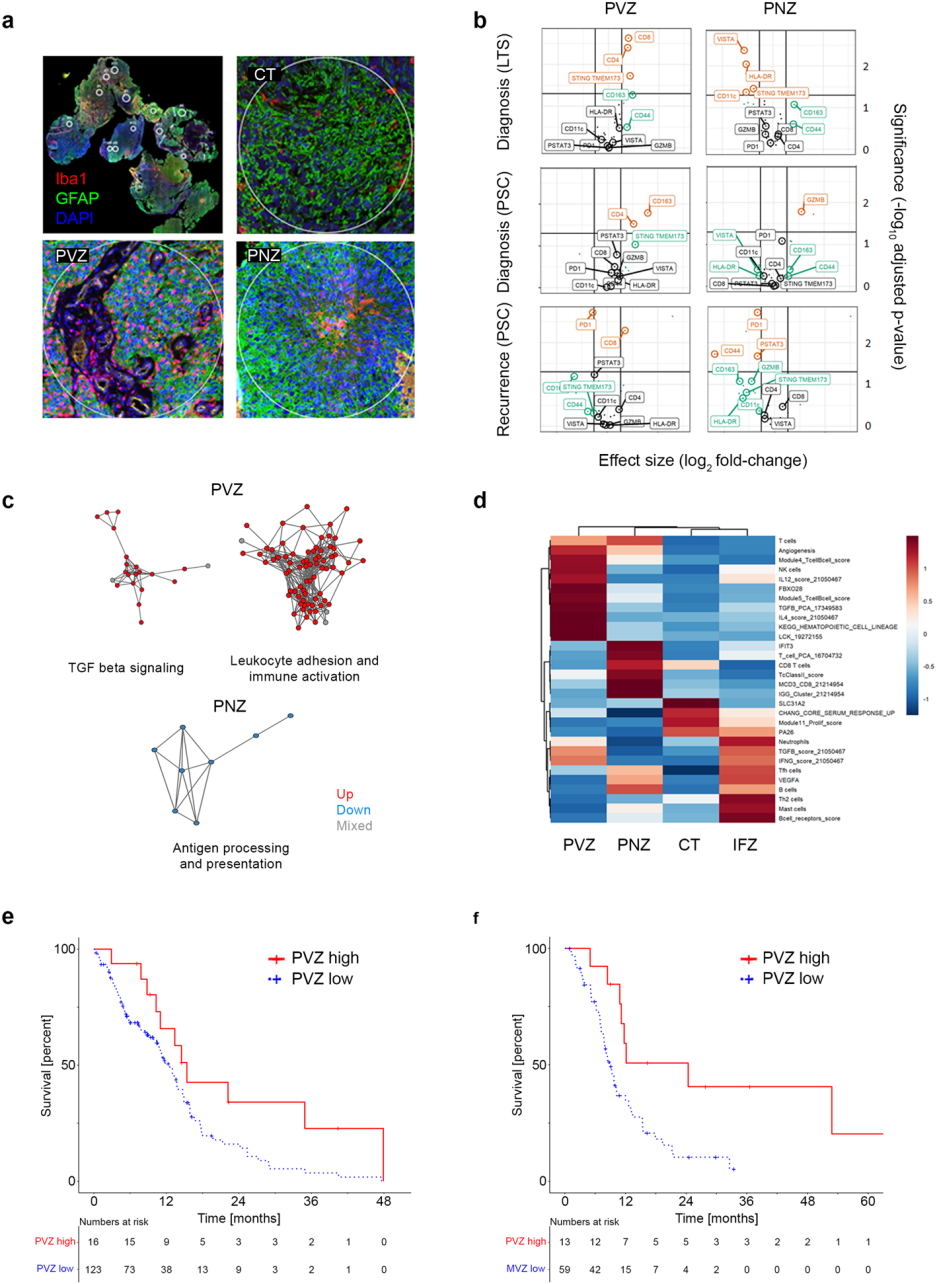
Spatial immune profiling of glioblastoma to predict survival. **a** Morphology-based selection of regions of interest following co-incubation of glioblastoma tissue sections with indicated fluorescence-markers and an immune-oncology panel of 28 antibodies tagged with UV cleavable DNA bar codes; *CT* cell-dense tumor; *PVZ* perivascular zone; *PNZ* perinecrotic zone. **b** Volcano plots indicating differential marker abundance in the PVZ and PNZ by clinical subgroups relative to CT; *LTS* long-term surviving patients; *PSC* paired-samples cohort. **c** Network analysis of differentially expressed GO gene sets in the Ivy GAP dataset; each node represents a gene set colored by the direction of enrichment of individual genes relative to CT (geneset expression: red, up; blue, down; gray, mixed); nodes are connected by edges if they share at least one gene. **d** Random forest classification of Ivy GAP tumor regions based on pan-immune gene sets; feature importance plot of the 20 most important gene sets; IFZ, infiltration zone. **e**, **f** Overall survival of patients with newly diagnosed glioblastoma (**e**) and post-recurrence survival of patients with recurrent glioblastoma (**f**) segregated by high versus low gene expression of the PVZ spatial immune profile

Enrichment of antibody-linked DNA barcodes in perivascular and perinecrotic compartments was analyzed utilizing cellular tumor regions as reference (Fig. 1b). In the perivascular zone of LTS, there was enrichment of CD8 (*p* = 0.002) and of the innate immunity activator, stimulator of interferon genes (STING, *p* = 0.019). By contrast, the immunosuppressive macrophage marker CD163 was enriched in the perivascular zone among newly diagnosed tumors from non-LTS patients (p = 0.016). In the perinecrotic zone of tumors of LTS, but not of non-LTS patients, reduced levels of the immune checkpoint molecule V-domain Ig suppressor of T-cell activation (VISTA, *p* = 0.004), and reduced levels of the pro-inflammatory markers STING (*p* = 0.035), CD11c (*p* = 0.043) and HLA-DR (*p* = 0.009) were noted. In matched recurrent versus primary tumors of non-LTS patients, there was an enrichment of the cytotoxic lymphocyte marker CD8 in the perivascular zone, and underrepresentation of the immune checkpoint molecule programmed cell death protein 1 throughout spatial compartments (Fig. 1b, Figure S2), supporting the previous reports of a more inflammatory immune phenotype at recurrence [4].

We expanded on these analyses by microenvironment-focused deconvolution of the Ivy Glioblastoma Atlas Project (GAP) spatial RNAseq dataset (https://glioblastoma.alleninstitute.org). CIBERSORT digital cytometry (https://cibersortx.stanford.edu) confirmed immunosuppressive macrophage accumulation in the perivascular zone (Figure S3). Gene ontology (GO) network analyses, depicting clusters of genesets with at least one overlapping gene as interconnected nodes, identified simultaneous enrichment of pro- and anti-inflammatory gene expression clusters in the perivascular zone, related to leukocyte adhesion and immune activation, and to transforming growth factor beta signaling, respectively, whereas in the perinecrotic zone, a GO network related to antigen processing and presentation was down-regulated (Fig. 1c).

Next, we sought to explore whether spatial immune profiles were associated with survival. For this purpose, we utilized the Ivy GAP dataset to develop a random forest-based classifier by employing an established pan-cancer immunity gene set panel (Fig. 1d). This classifier was then applied to bulk RNAseq data from two clinically annotated cohorts of patients with newly diagnosed or recurrent glioblastoma, followed by k-means clustering (Figure S4). The cellular tumor gene expression pattern was blended by this approach due to overlap with the perivascular, perinecrotic and infiltration zone patterns (Figure S5), thus circumventing sensitivity bias with respect to these less-abundant spatial compartments. We noted longer survival of glioblastoma patients with enrichment of the perivascular immunity gene expression pattern at diagnosis (*p* = 0.016, Fig. 1e) or at first recurrence (*p* = 0.012, Fig. 1f). By contrast, enrichment of the immune profile that classified the infiltration zone was associated with inferior survival in three independent cohorts of patients with recurrent glioblastoma (Figure S6), indicating a potential obstacle to immunotherapy design.

Our proof-of-concept analyses highlight clinical implications of spatial immune phenotypes in glioblastoma, including differential abundance of targetable cancer immune modulators (e.g., STING, VISTA) and associations of spatial immune profiles with outcome. Collectively, our findings support the notion that pharmacologic immune modulation could indeed be exploited for the benefit of glioblastoma patients, but suggest that a more granular, spatially resolved understanding of spatial immunity may be warranted to inform immunotherapy design.

## Supplementary Information

Below is the link to the electronic supplementary material.

Supplementary file1 (PDF 1928 kb)
