# Supplementary material for: Spatial immune profiling of glioblastoma identifies an inflammatory, perivascular phenotype associated with longer survival
Source: Acta Neuropathol. 2023 Aug 13;146(4):647–9. doi: 10.1007/s00401-023-02617-6 (PMC10499942; doi:10.1007/s00401-023-02617-6)
Supplement: Supplementary file 1 — Supplementary file1 (PDF 1928 kb) [file 401_2023_2617_MOESM1_ESM.pdf]

Spatial immune profiling of glioblastoma identifies an inflammatory, perivascular immune phenotype associated with longer survival

SUPPLEMENTARY MATERIAL

Note S1. Patients and methods……………….……….…………….………..………2

Note S2. Funding sources………………………...………………………………………6

Table S1. Digital spatial profiling antibody panel………………………………………7

Table S2. Patient characteristics………………….……………………………………..8

Figure S1. Overall survival of spatial immune profiling cohorts…………………………..9

Figure S2. Abundance of CD8 and PD1 in paired glioblastoma samples…………….10

Figure S3. Spatial digital cytometry………………………………….....……….....……11

Figure S4. Spatial immune profiling to predict patient survival…………………………12

Figure S5. Spatial eigengeneset signatures…….………………………………………13

Figure S6. Spatial immune profiling to predict patient survival.………………………...14

References……………………………………………………………………………..15

Note S1. Patients and methods.

Patient tissue samples

Formalin-fixed and paraffin-embedded tissue sections with confirmed diagnosis of an IDH wild-type glioblastoma according to the 2021 World Health Organization classification of central nervous system tumors [8] were utilized for GeoMx digital spatial immune profiling [9]. Sections were selected by a board-certified neuropathologist (JF) based on the presence of perivascular and perinecrotic compartments. Patients provided written informed consent for the use of their tissue samples and clinical data for research purposes. Matched primary and recurrent tissue samples were derived from the central nervous system tumor tissue bank Düsseldorf from a previous research project that was approved by the Ethics Committee of the Medical Faculty at Heinrich Heine University Düsseldorf (study number 4940, [7]). Tissue samples of long-term surviving patients were derived from the EORTC 1419 study, which was approved by the ethics board of the Canton of Zurich (study number KEK 2014-0555) and locally at each participating site [4].

GeoMx Digital Spatial Profiling

Formalin-fixed and paraffin-embedded tissue sections were co-incubated with 28 antibodies labeled with photocleavable DNA bar codes for immune profiling (Table S1, Nanostring Inc, Seattle, WA, USA) and with three fluorescence-labelled antibodies to visualize tissue morphology, including murine anti-glial fibrillary acidic protein (GFAP, clone 5C10, Novus Biologicals, Littleton, CO, USA) for tumor cells, anti-Iba1 for myeloid cells (clone E4O4W, Cell Signaling Technology, Danvers, MA, USA) and anti-CD31 for endothelial cells (clone JC/70A, Abcam, Cambridge, UK), as well as 4,6-

Diamidin-2-phenylindol (DAPI) to stain cell nuclei through DNA intercalation (Thermo Fisher Scientific, Waltham, MA, USA).

Slides were then scanned utilizing a GeoMx Digital Spatial Profiler (Nanostring Inc) and regions of interest selected remotely by a board-certified neuropathologist (JF). Subsequently, automated photocleavage and aspiration of DNA bar codes was performed sequentially for each region of interest. DNA bar codes were then quantified utilizing an nCounter Sprint Profiler (Nanostring Inc). DNA bar code counts were normalized to External RNA Control Consortium (ERCC) spike-in controls and housekeeping genes, and were corrected with respect to donor and batch. No per-gene standardization was performed, except for the extra correction for CD45 expression for each sample. Per tumor sample, spatial expression values were averaged between regions of interest for each histopathologic compartment, i.e. cellular tumor, perivascular and perinecrotic zones.

RNA sequencing datasets

The Ivy glioblastoma atlas project (GAP) spatial RNA sequencing core dataset, comprising 120 spatial transcriptomes from 10 patients with IDH wild-type glioblastoma [13] was utilized for gene ontology (GO) network analyses of differentially expressed immunity genesets (Figure 1c) and for the development of a spatial immunity classifier (Figure 1d). The spatial immunity classifier was then applied to bulk RNA sequencing datasets of IDH wild-type gliobastoma samples, including N=139 newly diagnosed glioblastomas from The Cancer Genome Atlas pan-cancer study [5] (Figure 1e), a merged dataset of N=72 recurrent glioblastomas [14] (Figure 1f, Figure S6a) as well as two smaller studies comprising N=36 [7] (Figure S6b) and N=29 [2] (Figure S6c) recurrent glioblastomas, respectively.

Geneset network analyses

Geneset network analyses were conducted in the Ivy GAP dataset to identify differentially expressed genesets in the perivascular zone versus cellular tumor and in the perinecrotic zone versus cellular tumor, respectively (Figure 1c [11, 12]). First, differential expression of GO genesets [1, 3] with a size < 200 genes was analyzed between two groups of interest. Genesets with a false discovery rate < 0.05 were then utilized for network discovery by depicting differentially expressed genesets as nodes that were connected by edges if they shared at least one gene. Genesets that were up-regulated in the perivascular or perinecrotic zone compared to cellular tumor were colored red, genesets that were down-regulated were colored blue, and genesets in which some genes were up- and others down-regulated were colored gray. Doublets and community-connecting edges were removed to split large clusters into smaller ones based on edge betweenness. Gene set names were split into individual words and counted to automatically construct cluster labels.

Classification of spatial immunity gene expression patterns

A random forest algorithm to discriminate spatial gene expression patterns based on N=72 pan-caner immunity-associated gene sets [13] was trained on N=80 randomly selected spatial RNAseq samples from the Ivy GAP dataset and was tested on N=40 samples from the same dataset [12]. Weighted correlation network analysis delivered 3 coexpression clusters of immune genesets characterizing the different glioma regions (eigengenesets). Clustering of all N=120 spatial RNAseq samples based on the expression of these eigengenesets revealed associations with the perivascular, perinecrotic and infiltration zone samples, whereas cellular tumor was characterized by lack of expression of these clusters (Figure S5). Eigengeneset clustering indicated overlap of the cellular tumor expression pattern with the other three patterns. The GO

terms constituting eigengeneset clusters were imputed to the CIBERSORT online tool to classify bulk RNAseq datasets (Figure S4). On note, the lack of genesets specific for classifying cellular tumor led to an intended focus on the relative distribution of perivascular, perinecrotic and infiltration zone immunity patterns. No further filtering methods were applied for this purpose.

The CIBERSORT digital cytometry online classification tool [9] was also used for immune cell estimates in RNAseq datasets, including (i) the spatial gene expression dataset from Ivy GAP (Figure S3, [12]), (ii) the newly diagnosed glioblastoma bulk gene expression dataset from TCGA (Figure 4a, [5]) and a merged dataset of recurrent glioblastomas (Figure 4b, [14]).

Survival analyses

Survival analyses were performed in R using the *coxph* function from the survival package (Version 3.4.0). Scores from the random forest-based classifier were used to automatically devise a cutoff using the *surv_cutpoint* function from the survminer package (Version 0.4.9) and then used as input for the *coxph* function. For the newly diagnosed glioblastoma cohort [3], *MGMT* promoter methylation status was also used as input, whereas limited availability of *MGMT* promoter methylation status precluded respective adjustments in the recurrent glioblastoma cohorts [2, 7, 14].

Biostatistics tools

All statistical analysis was performed using the R environment for statistical computing (R version 3.5.1, 2018-07-02) with Bioconductor [6] and dedicated packages. All analysis and this report is produced programatically using R markdown (https://CRAN.R-project.org/package=rmarkdown) in Rstudio (RStudio Inc, Boston, MA, USA), compliant with the principles of Reproducible Research [10].

Note S2. Funding sources.

This study was funded by grants from the OPO foundation, the Desirée und Niels Yde foundation, Charlotte and Nelly Dornacher Foundation, the Swiss National Science Foundation (P2SKP3-158656) and Oncosuisse (KLS-4870-08-2019) to HGW, by a grant of the University of Zurich (UZH) Foundation to MW and by the Clinical Research Priority Program (CRPP) of the University of Zurich for the CRPP ImmunoCure to PR. The EORTC 1419 ETERNITY study was supported by a generous grant from the Brain Tumor Funders' Collaborative (American Brain Tumor Association, Brain Tumour Foundation of Canada, James S. McDonnell Foundation, Childrens` Brain Tumor Foundation, The Sontag Foundation) and by the EORTC Brain Tumor Group.

Table S1. Digital spatial profiling antibody panel.

CD45	Bone marrow derived cells
CD3	T cells
CD4	T helper cells
CD8	Cytotoxic T cells
CD45RO	Memory T cells
FoxP3	Regulatory T cells
CD20	B cells
CD56	NK cells
CD68	Monocytes, macrophages
CD44	Pro-inflammatory macrophages
CD163	Immunosuppressive macrophages
HLA-DR	Antigen-presenting cells
CD11c	Antigen-presenting cells
CD66b	Hematopoietic stem cells
GZMB	cytotoxicity
STAT3 (pY705)	Pro-inflammatory signaling programs
4-1BB	Immune checkpoint
B7-H3	Immune checkpoint
B7-H4	Immune checkpoint
ICOS	Immune checkpoint
IDO1	Immune checkpoint
OX40L	Immune checkpoint
PD1	Immune checkpoint
PD-L1	Immune checkpoint
STING	Immune checkpoint
VISTA	Immune checkpoint
S100B	House keeping
S6	House keeping

Table S2. Patient characteristics.

	All patients (N=20)	Paired samples cohort (N=10)	Long-term survivors (N=10)	p-value PSC vs LTS
Age at diagnosis:<br>Median (range)	61 (14-72)	62 (14-72)	59 (49-68)	.97
Gender: N (%)<br>female<br>male	 6 (30)<br>14 (70)	 3 (30)<br>7 (70)	 3 (30)<br>7 (70)	 <br>1.00
Extent of resection: N (%)<br>Gross total<br>Partial<br>No data	 15 (83)<br>3 (17)<br>2	 7 (78)<br>2 (22)<br>1	 8 (89)<br>1 (11)<br>1	 <br>1.00
MGMT promoter status: N (%)<br>Methylated<br>Unmethylated	 11 (55)<br>9 (45)	 3 (30)<br>7 (70)	 8 (80)<br>2 (20)	 <br>0.025

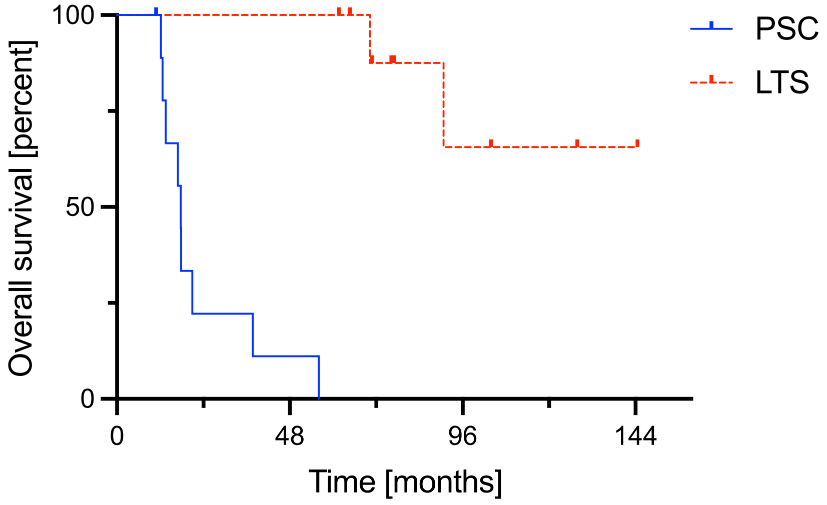

Figure S1. Overall survival of spatial immune profiling cohorts of patients with glioblastoma. PSC, paired sample cohort; LTS, long-term survivor cohort.

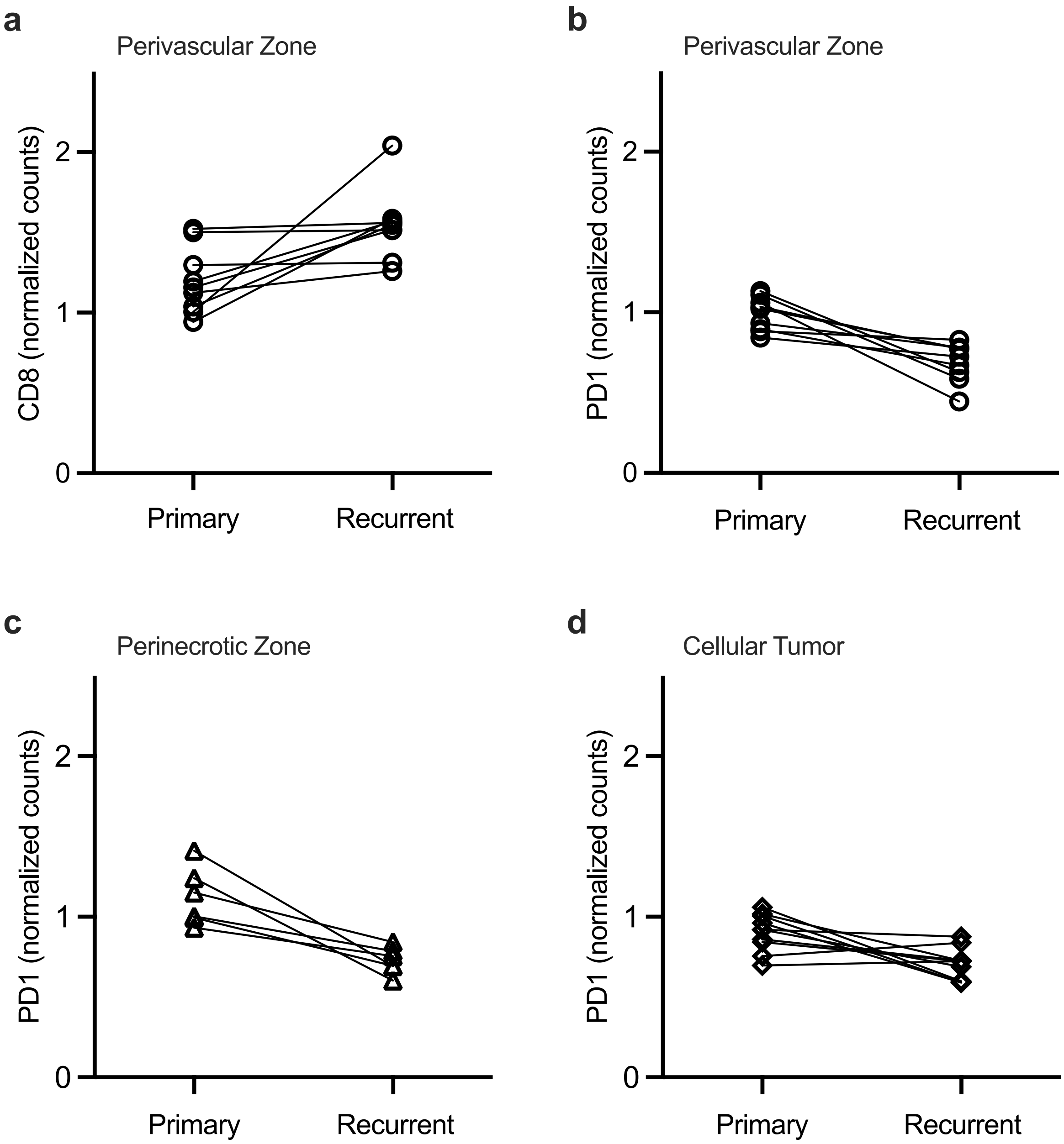

Figure S2. Abundance of CD8 and PD1 in paired primary and recurrent glioblastoma samples. Protein abundance was assessed by GeoMx digital spatial profiling in the PCS cohort. Normalized counts for each protein were averaged over regions of interest individually for each sample.

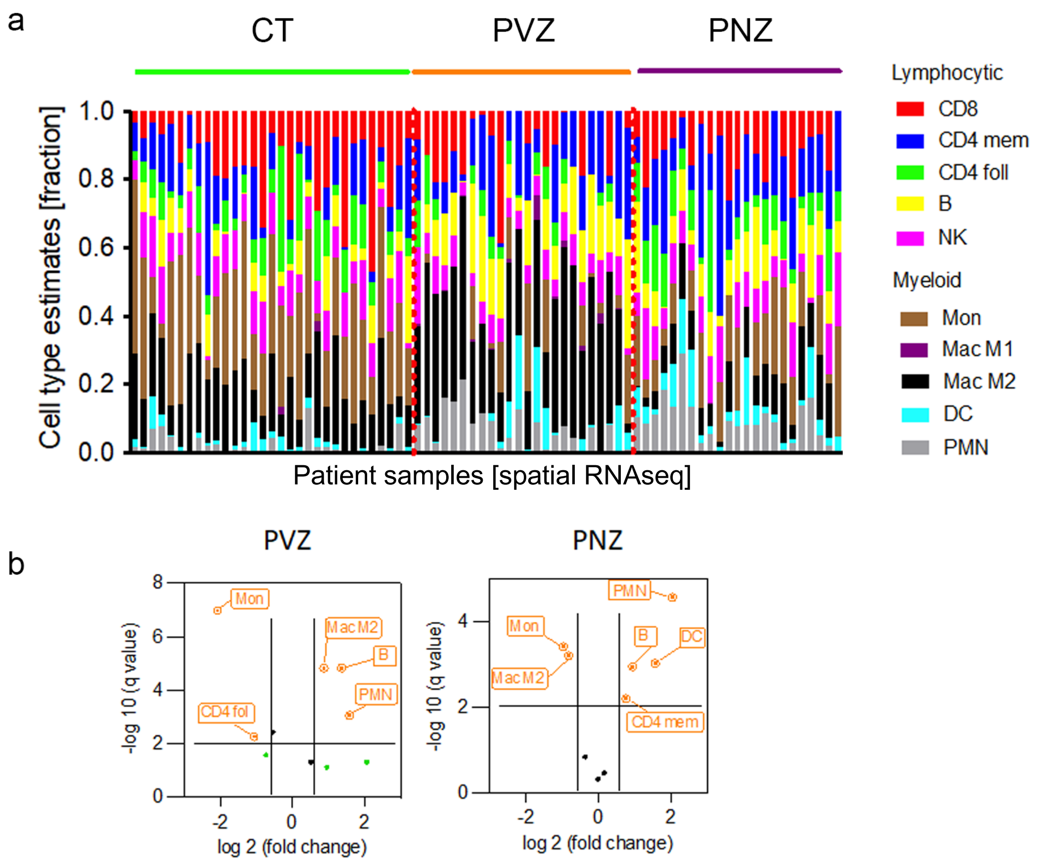

Figure S3. Spatial digital cytometry. a, immune cell type abundance in the Ivy GAP dataset [12] was estimated utilizing the CIBERSORT digital cytometry algorithm [9]. **b,** volcano plot depicting cell type abundance in the perivascular zone (PVZ) and perinecrotic zone (PNZ) relative to cellular tumor regions (CT). CD8, cytotoxic T cells; CD4 mem, memory T helper cells; CD4 foll, follicular T helper cells; B, B cells; NK, natural killer cells; Mon, monocytes; Mac M1, pro-inflammatory macrophages; Mac M2, immunosuppressive macrophages; DC, dendritic cells; PMN, polymorphonuclear leukocytes.

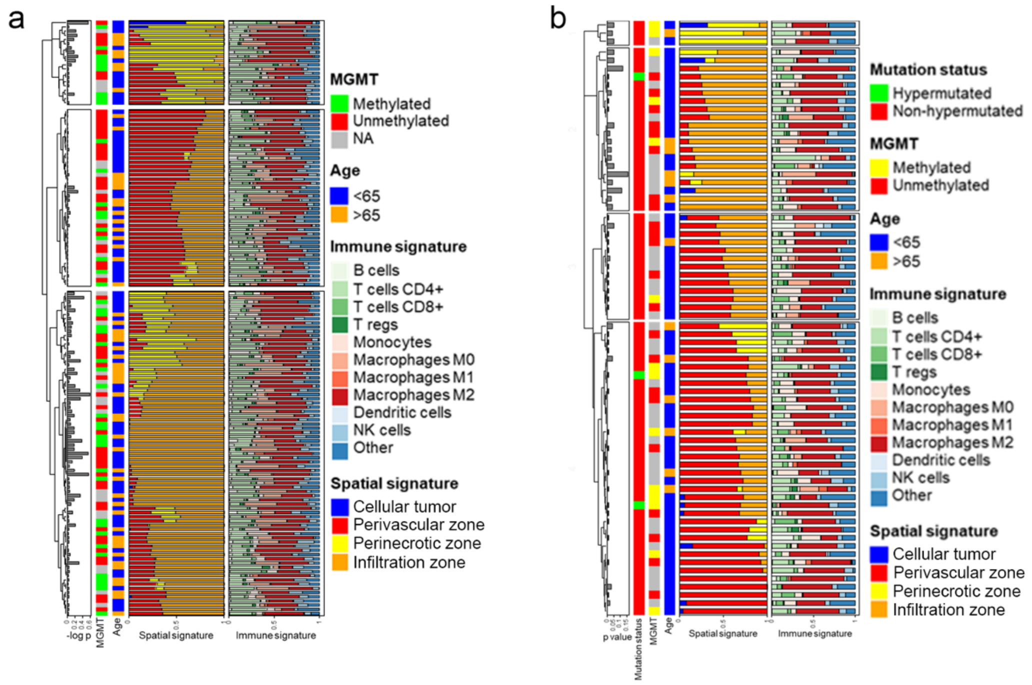

Figure S4. Spatial immune classification of bulk tumor samples. Patients with newly diagnosed glioblastoma (**a**, N=139, Ref. [5]) and with recurrent glioblastoma (**b,** N=72, Ref. [14]) were clustered by abundance of a random-forest based spatial gene expression signature that was based on pan immune gene sets (Figure 1D, Ref. [13]). Spatial signatures and digital cytometry immune signatures were annotated utilizing the CIBERSORT classifying tool [9].

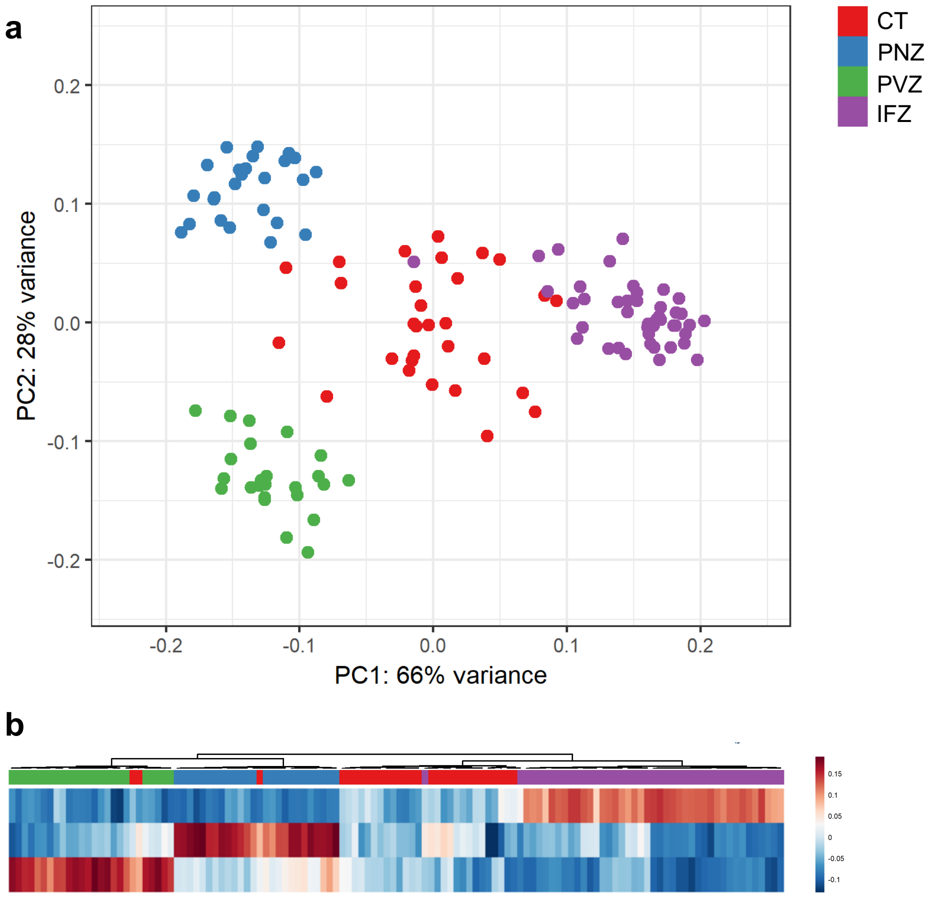

Figure S5. Spatial eigengeneset signatures. A random forest immunity classifier was trained utilizing pan-cancer immunity genesets [13] in the Ivy GAP spatial RNAseq dataset [12]. Coexpression clusters of GO-terms characterizing the different glioma regions (eigengenesets) were employed to test all samples of the Ivy GAP dataset. **a,** principal component analysis plot based on the eigengeneset signature. **b,** eigengeneset signature of all samples based on geneset clusters.

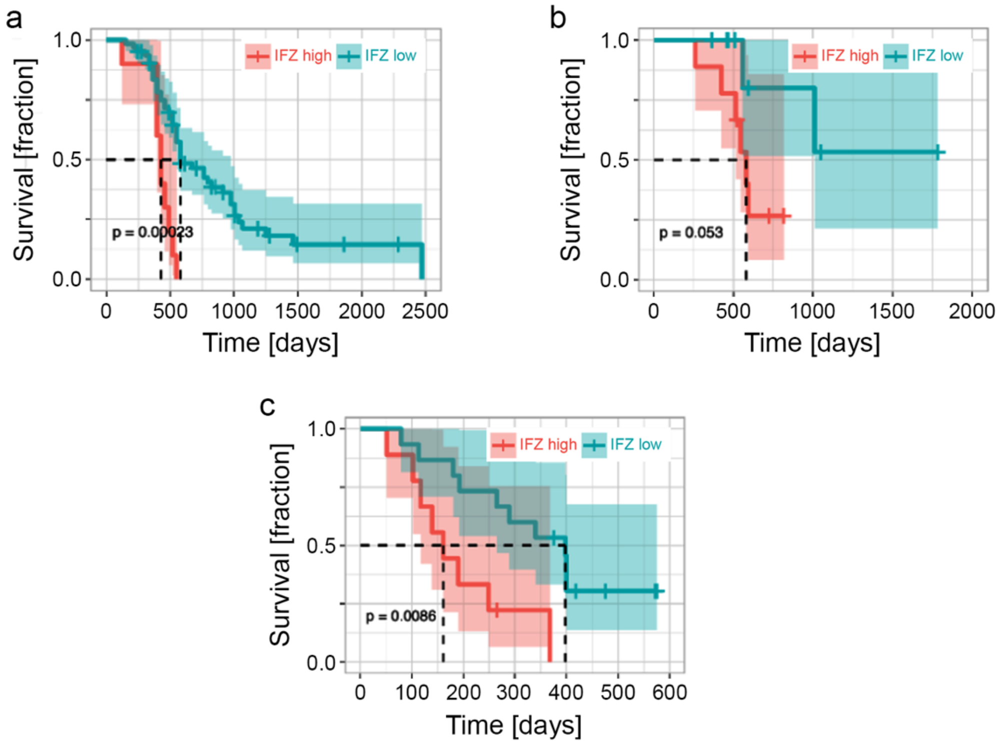

Figure S6. Spatial immune profiling to predict patient survival. Survival of patients with recurrent glioblastoma following classification by Ivy GAP spatial immune profiles; CIBERSORT was utilized for classifier application and a cut-off of 0.5 was employed to define infiltration zone (IFZ) high vs low scores; the logrank test was utilized for curve comparison; datasets: **a**, ref. [14], N=72; **b**, ref. [7], N=36; **c**, ref. [2], N=29.

References

1 Ashburner M, Ball CA, Blake JA, Botstein D, Butler H, Cherry JM, Davis AP, Dolinski K, Dwight SS, Eppig JT et al (2000) Gene ontology: tool for the unification of biology. The Gene Ontology Consortium. Nat Genet 25: 25-29 Doi 10.1038/75556

2 Cloughesy TF, Mochizuki AY, Orpilla JR, Hugo W, Lee AH, Davidson TB, Wang AC, Ellingson BM, Rytlewski JA, Sanders CM et al (2019) Neoadjuvant anti-PD-1 immunotherapy promotes a survival benefit with intratumoral and systemic immune responses in recurrent glioblastoma. Nat Med 25: 477-486 Doi 10.1038/s41591-018-0337-7

3 Gene Ontology C, Aleksander SA, Balhoff J, Carbon S, Cherry JM, Drabkin HJ, Ebert D, Feuermann M, Gaudet P, Harris NL et al (2023) The Gene Ontology knowledgebase in 2023. Genetics 224: Doi 10.1093/genetics/iyad031

4 Hertler C, Felsberg J, Gramatzki D, Le Rhun E, Clarke J, Soffietti R, Wick W, Chinot O, Ducray F, Roth P et al (2023) Long-term survival with IDH wildtype glioblastoma: first results from the ETERNITY Brain Tumor Funders' Collaborative Consortium (EORTC 1419). Eur J Cancer 189: 112913 Doi 10.1016/j.ejca.2023.05.002

5 Hoadley KA, Yau C, Hinoue T, Wolf DM, Lazar AJ, Drill E, Shen R, Taylor AM, Cherniack AD, Thorsson V et al (2018) Cell-of-Origin Patterns Dominate the Molecular Classification of 10,000 Tumors from 33 Types of Cancer. Cell 173: 291-304 e296 Doi 10.1016/j.cell.2018.03.022

6 Huber W, Carey VJ, Gentleman R, Anders S, Carlson M, Carvalho BS, Bravo HC, Davis S, Gatto L, Girke T et al (2015) Orchestrating high-throughput genomic analysis with Bioconductor. Nat Methods 12: 115-121 Doi 10.1038/nmeth.3252

7 Korber V, Yang J, Barah P, Wu Y, Stichel D, Gu Z, Fletcher MNC, Jones D, Hentschel B, Lamszus K et al (2019) Evolutionary Trajectories of IDH(WT) Glioblastomas Reveal a Common Path of Early Tumorigenesis Instigated Years ahead of Initial Diagnosis. Cancer Cell 35: 692-704 e612 Doi 10.1016/j.ccell.2019.02.007

8 Louis DN, Perry A, Wesseling P, Brat DJ, Cree IA, Figarella-Branger D, Hawkins C, Ng HK, Pfister SM, Reifenberger G et al (2021) The 2021 WHO Classification of Tumors of the Central Nervous System: a summary. Neuro Oncol 23: 1231-1251 Doi 10.1093/neuonc/noab106

9 Newman AM, Steen CB, Liu CL, Gentles AJ, Chaudhuri AA, Scherer F, Khodadoust MS, Esfahani MS, Luca BA, Steiner D et al (2019) Determining cell type abundance and expression from bulk tissues with digital cytometry. Nat Biotechnol 37: 773-782 Doi 10.1038/s41587-019-0114-2

10 Peng RD (2009) Reproducible research and Biostatistics. Biostatistics 10: 405-408 Doi 10.1093/biostatistics/kxp014

11 Prummer M (2019) Enhancing gene set enrichment using networks. F1000Res 8: 129 Doi 10.12688/f1000research.17824.2

12 Puchalski RB, Shah N, Miller J, Dalley R, Nomura SR, Yoon JG, Smith KA, Lankerovich M, Bertagnolli D, Bickley K et al (2018) An anatomic transcriptional atlas of human glioblastoma. Science 360: 660-663 Doi 10.1126/science.aaf2666

13 Thorsson V, Gibbs DL, Brown SD, Wolf D, Bortone DS, Ou Yang TH, Porta-Pardo E, Gao GF, Plaisier CL, Eddy JA et al (2018) The Immune Landscape of Cancer. Immunity 48: 812-830 e814 Doi 10.1016/j.immuni.2018.03.023

14 Wang J, Cazzato E, Ladewig E, Frattini V, Rosenbloom DI, Zairis S, Abate F, Liu Z, Elliott O, Shin YJ et al (2016) Clonal evolution of glioblastoma under therapy. Nat Genet 48: 768-776 Doi 10.1038/ng.3590
